# *Staphylococcus aureus* Floating Biofilm Formation and Phenotype in Synovial Fluid Depends on Albumin, Fibrinogen, and Hyaluronic Acid

**DOI:** 10.3389/fmicb.2021.655873

**Published:** 2021-04-29

**Authors:** Samantha Knott, Dylan Curry, Neil Zhao, Pallavi Metgud, Sana S. Dastgheyb, Caroline Purtill, Marc Harwood, Antonia F. Chen, Thomas P. Schaer, Michael Otto, Noreen J. Hickok

**Affiliations:** ^1^Department of Orthopaedic Surgery, Sidney Kimmel Medical College of Thomas Jefferson University, Philadelphia, PA, United States; ^2^Rothman Orthopaedic Institute, Philadelphia, PA, United States; ^3^Department of Orthopaedic Surgery, Brigham and Women’s Hospital, Boston, PA, United States; ^4^Department of Clinical Studies, New Bolton Center, School of Veterinary Medicine, University of Pennsylvania, Kennett Square, PA, United States; ^5^Pathogen Molecular Genetics Section, Laboratory of Human Bacterial Pathogenesis, National Institute of Allergy and Infectious Diseases, National Institutes of Health, Bethesda, MD, United States

**Keywords:** *Staphylococcus aureus*, biofilm, synovial fluid, antibiotic tolerance, virulence factors

## Abstract

Biofilms are typically studied in bacterial media that allow the study of important properties such as bacterial growth. However, the results obtained in such media cannot take into account the bacterial localization/clustering caused by bacteria–protein interactions *in vivo* and the accompanying alterations in phenotype, virulence factor production, and ultimately antibiotic tolerance. We and others have reported that methicillin-resistant or methicillin-susceptible *Staphylococcus aureus* (MRSA or MSSA, respectively) and other pathogens assemble a proteinaceous matrix in synovial fluid. This proteinaceous bacterial aggregate is coated by a polysaccharide matrix as is characteristic of biofilms. In this study, we identify proteins important for this aggregation and determine the concentration ranges of these proteins that can reproduce bacterial aggregation. We then test this protein combination for its ability to cause marked aggregation, antibacterial tolerance, preservation of morphology, and expression of the phenol-soluble modulin (PSM) virulence factors. In the process, we create a viscous fluid that models bacterial behavior in synovial fluid. We suggest that our findings and, by extension, use of this fluid can help to better model bacterial behavior of new antimicrobial therapies, as well as serve as a starting point to study host protein–bacteria interactions characteristic of physiological fluids.

## Introduction

Despite aggressive treatment and the use of local supratherapeutic concentration of antimicrobials, joint infections can persist (Kunutsor et al., 2018), even in the absence of implants. The presence of synovial fluid in the joint fosters the formation of dense, proteinaceous, free-floating biofilm aggregates (Dastgheyb et al., 2015a,b; Crosby et al., 2016; Gilbertie et al., 2019; Pestrak et al., 2020), the removal of which is required for successful treatment of infection. The bacteria–protein interactions in these aggregates induce alterations in phenotype, virulence factor production, and ultimately marked antibiotic tolerance (Dastgheyb et al., 2015a,b,c), akin to what has been described for adherent biofilms.

Under conditions that mimic the physiological environment, bacterial aggregation also occurs *in vitro*, where microscopic clusters are seen in serum and microscopic and macroscopic clusters are visible in synovial fluid (Dastgheyb et al., 2015a; Crosby et al., 2016). We and others have reported that in synovial fluid, methicillin-resistant or methicillin-susceptible *Staphylococcus aureus* (MRSA or MSSA, respectively) and other pathogens assemble a proteinaceous matrix that is coated by polysaccharides as is characteristic of biofilms (Dastgheyb et al., 2015b; Perez and Patel, 2015; Gilbertie et al., 2019). Aggregation depends on microbial surface components recognizing adhesive matrix molecules (MSCRAMMs) and the master biofilm regulator accessory gene regulator (Agr) (Dastgheyb et al., 2015b). Agr-regulated virulence factors, specifically phenol-soluble modulins (PSMs), are downregulated in synovial fluid. This downregulation appears to have consequences on bacterial aggregation, as we found that overexpression of any type of PSM (PSMα, PSMβ, and delta toxin) abrogated synovial fluid-induced aggregation. These findings support the notion that suppression of Agr and the concomitant strong suppression of PSM production are critical for MRSA aggregation (Dastgheyb et al., 2015c). Thus, these reports challenge whether drugs that act through inhibition of *agr*-mediated gene expression, such as production of proteins important for quorum sensing, will result in a benefit to the host during periprosthetic joint infection (PJI) (Piewngam et al., 2020). In light of these findings, we reasoned that it would be important to begin mapping the components of these aggregates to allow better modeling both for implant development as well as for combatting joint infection.

Synovial fluid is central to aggregation and is a protein-rich viscous fluid composed of proteins filtered from blood and secreted proteins/proteoglycans from cells (predominantly synovium and cartilage). In our studies, we have recognized that synovial fluid shows donor variability, perhaps where composition reflects disease state (Mateos et al., 2012; Noh et al., 2014). To circumvent this limitation, we have established equine or porcine synovial fluid as a readily available source of “normal” synovial fluid that reproduces the reported aggregatory behavior and phenotype over multiple bacterial species (Gilbertie et al., 2019).

In this study, we set out to determine which proteins in synovial fluid were predominantly responsible for staphylococcal aggregation. To do that, the abundant proteins sequestered in synovial fluid bacterial aggregates were identified and their roles were investigated. Based on these determinations, we next evaluated if the presence of these proteins/proteoglycans could reproduce the phenotype of synovial fluid bacterial aggregates, where our criteria were (1) morphology, (2) composition, (3) antimicrobial tolerance, and (4) virulence factor production. As a consequence of these studies, we have developed a “pseudo” synovial fluid (pSynF) that embodies many of the characteristics of native synovial fluid and serves as a readily accessible means to study bacterial behavior in fluids that more closely approximate *in vivo* biofilm characteristics.

## Materials and Methods

### Ethics Statement

Human synovial fluid and other surgical waste were obtained from therapeutically necessary joint aspirations or operations. This material, designated as “waste” (no identifiers), was retrieved and designated as “not human research” by the Thomas Jefferson University Office of Human Research Protections in keeping with the revised Federal Policy for the Protection of Human Subjects (revised Common Rule, 2018).

### Bacterial Strains and Growth

MSSA ATCC ^®^25923^TM^ and MRSA LAC USA300 [parent strain, giving rise to LAC *psm*α-lux, LAC *psm*β-lux, or LAC P3-lux strains (Dastgheyb et al., 2015c)] were grown from a single colony in Trypticase Soy Broth (TSB; Becton-Dickinson, Sparks, MD) overnight (ON), 37°C, 180 rpm. ON cultures were diluted by comparison to a 0.5 McFarland standard [∼10^8^ colony-forming unit (CFU)/ml for *S. aureus*].

### Clinical Samples

Except for the PJI samples, synovial fluid used in this manuscript was collected as discarded material from routine office aspirations from patients with sterile fluid excess. Eight of those were used without pooling for determination of MSSA aggregate characteristics by sodium dodecyl sulfate–polyacrylamide gel electrophoresis (SDS-PAGE) and Western blotting. Otherwise, 2–5 samples were pooled to allow volumes large enough to perform the various activity/expression assays throughout the rest of the manuscript. The infected samples were collected as waste from revision surgeries for treatment of confirmed PJI and represented implant-adherent biofilm and non-attached biofilm. A small sample was scraped from the implant (biofilm sample), and another sample was retrieved from the discarded fluid material that was flushed from the bone cavity (floating aggregate). Material was placed into labeled 50-ml conical tubes, fixed by incubation in formalin, and, after rinsing, prepared for imaging by scanning electron microscopy (SEM). The identity of the bacterial strain causing PJI was not supplied with the fixed samples.

### Biofilm Formation

To form biofilms *in vitro*, MSSA (10^5^ CFU/ml) were incubated in TSB or human synovial fluid for 24 h, 37°C, without agitation using 1 cm × 2 mm Ti6Al4V coupons (kind gift of Zimmer). Surfaces were gently rinsed 3 × with phosphate buffered saline (PBS) and fixed with 4% paraformaldehyde, ON, 4°C. Samples were then prepared for SEM. Clinical examples were composed of samples of infected reamings from a PJI patient as well as several scrapings from the implant removed during the patient’s treatment. From this formalin-fixed material, at least three samples were removed and rinsed and solid material was affixed to a slide followed by dehydration, sputter coating, and SEM analysis. Micrographs were compared for fibrous material, presence of blood cells, and apparent biofilm-like structures that could be attributed to bacteria. Images representative of these are shown.

### Determination of Interplay of Synovial Fluid Components on Methicillin-Susceptible *Staphylococcus aureus* Aggregation and Formation of Pseudo Synovial Fluid

Hyaluronic acid (HA; *Streptococcus equi*, 91% high molecular weight (MW); Alfar Aesar, Tewksbury, MA) was dissolved (ON, 4°C) to 4.05 mg/ml in sterile TSB. Fibrinogen (Fg; Alfa Aesar, Tewksbury, MA, United States) was dissolved to 60, 90, and 120 mg/ml in PBS, pH 8.5, by incubation at 37°C, 3 min, followed by vortexing. Albumin (Alb; Fisher Scientific, Pittsburgh, PA, United States) was dissolved to 60, 120, and 180 mg/ml in PBS, pH 8.5. All stocks were sterile filtered (0.2 μm filter) and stored at 4°C for up to 2 days.

A concentration matrix of Fg, Alb, and HA was created in 96-well plates. TSB (140 μl) containing 0, 1.5, or 3.0 mg/ml HA was added to each well; 20 μl of Fg stocks were added to give 0, 6, 9, or 12 mg/ml and 20 μl of Alb stocks to yield 0, 6, 12, and 18 mg/ml. Lastly, 20 μl of 10^8^ CFU/ml MSSA in sterile PBS were added into each well to yield 10^7^ CFU/ml. Plates were incubated, 37°C, 180 rpm, 90 min to allow bacterial aggregation. Samples were serially diluted, followed by plating on Petrifilms; reduced bacterial counts were considered aggregation events (1 CFU would equal one aggregate in that instance). Each individual condition was determined in triplicate, with three independent experiments to yield an *n* = 8 or 9 for each condition. The combination of 3.0 mg/ml HA, 9 mg/ml Fg, and 10 mg/ml Alb in TSB was referred to as pSynF.

### Bacterial Aggregate Formation and Antibiotic Treatment

Pooled synovial fluid was formed by combining 2–5 samples from individual donors to better allow reproducibility. Here, 0.2, 1.0, or 3.0 ml of human synovial fluid (≥90% final volume), pSynF, human serum (Sigma, not heat inactivated) or TSB in 6-, 24-, or 96-well tissue culture plates (Med Supply Partners, Atlanta, GA), respectively, were inoculated with 10^7^ or 10^8^ CFU/ml MSSA or MRSA and incubated for a minimum of 90 min, 37°C, 125 rpm. Either during or after aggregation, amikacin (AMK) was added (0–200 μg/ml) and incubated for 6 h. At 6 h, bacteria were pelleted and washed [when aggregate dispersal was used, the resulting pellet was treated with 1 ml of 0.25% trypsin (Corning), 37°C, 180 rpm, 20 min, and pelleted again]. All pellets were then washed with PBS, resuspended by pipetting/vortexing and, after serial dilution, plated on 3M^TM^ Petrifilm^TM^ aerobic count plates (3M Corporation, St. Paul, MN), followed by direct counting (countable range, 30–300 CFU/spot). This dispersal method was validated by the (1) disappearance of visible aggregates and (2) equivalence of CFU (after Trypsin) with CFU recovered from TSB using an equal bacterial inoculum.

### Scanning Electron Microscopy

Bacterial aggregates were fixed with 4% paraformaldehyde, room temperature (RT), 10 min, and dehydrated by sequential incubation (RT, 10 min) with 10, 30, 50, 70, 90, and 100% ethanol in PBS. Samples were dried ON, sputter-coated with gold, and imaged using a Hitachi TM-1000 SEM.

### Fractionation by Sodium Dodecyl Sulfate–Polyacrylamide Gel Electrophoresis: Pseudo Synovial Fluid or Human Synovial Fluid

Synovial fluid samples were obtained from individual donors (*n* = 8), each separately labeled with a different number. Before fractionation, samples were diluted to 3% with sterile PBS. Aggregates formed as above were pelleted at 13,000 × g, 3 min, washed, and resuspended in 1 ml PBS by repeated vortexing and pipetting. Laemmli sample buffer with β-mercaptoethanol was added (1:1), samples heated at 70°C for 10 min, placed on ice, and fractionated (105 V, ∼70 min) on an SDS-polyacrylamide gel (7.5%), using the discontinuous buffer system of Laemmli (1970). MW standards (10–250 kDa; Precision Plus Protein^TM^ Dual Color Standards, Bio-Rad, Hercules, CA, United States) were run on the same gel, as were serum, Fg (Alfa Aesar), and Alb (Thermo Fisher Scientific).

### Western Blotting

Following fractionation, protein bands were transferred (1.3 A, 25V, 7 min) to nitrocellulose membranes (Bio-Rad) using the Trans Blot Turbo Transfer System (BioRad) with Turbo transfer buffer. Membranes were blocked by incubation in 10% nonfat milk (BioRad) in Tris-Buffered Saline (1×) containing 0.5% Tween-20 (10% TTBS), 60 min, followed by incubation with mouse anti-Fg (1:1,000; Sigma-Aldrich F9902) or rabbit anti-Alb (1:1,000; Sigma-Aldrich, SAB4200656) antibody, in 5% TTBS, 4°C, ON, with shaking. The blot was then washed 3× with 10% TTBS, incubated in horseradish peroxidase (HRP)-linked goat anti-mouse (Bio-Rad; 1:10,000) or HRP-linked goat anti-rabbit antibody (Abcam, Cambridge, MA; 1:20,000), RT, 2 h, washed 3×, followed by visualization using Pierce^TM^ ECL Western Blotting Substrate (Thermo Fisher Scientific, Rockford, IL, United States). In all gels and blots, results were repeated using additional aggregates formed in the same or different synovial fluid sample with similar appearances. Densitometry was performed using three different exposures of each blot. The amount of Fg in samples was calculated by comparison to the band area in 100 μg Fg control for each of the exposures.

### Synovial Fluid Fractionation

Pooled synovial fluid was formed by combining 2–5 samples from individual donors to better allow reproducibility. Protein was precipitated from pooled human synovial fluid (Qiu et al., 2003) by addition of 200 proof (100%) absolute ethanol, 4°C, to a final concentration of 10%, followed by ON incubation, 4°C. Protein precipitates were collected, 1,100 × g, 5 min, 4°C, where the pellet represented a Fg-enriched fraction (ppt) and the supernatant, a Fg-depleted synovial fluid (depSynF). SDS-PAGE and Western blotting were used to determine the extent of Fg depletion. Upon Fg re-addition, the concentrations added were 6, 9, and 12 mg/ml.

### Viscosity Determination

The viscosity of pooled synovial fluid and pSynF was measured by a homemade viscometer (Tang, 2016) using the following equation:

$$\eta =\frac{d^{2}\,\left(\rho_{b}-\rho_{l}\right)\,g\,\sin\theta }{18\,L}\,T$$

*T* = time (s) for a 2-mm diameter (*d*) ball bearing to traverse 0.1 m (*L*) of liquid in a 2-ml serological pipette. η = viscosity, ρ_b_ = 5156.6 kg/m^3^ = ball density; ρ_l_ = 1,000, 1,002, or 1,034 kg/m^3^, which are the density of water, synovial fluid, or pSynF, respectively; g = 9.8 m/s^2^ = gravitational acceleration; θ = π/3 radians = angle of the pipette with respect to the horizontal. Finally, all values were adjusted by multiplied by the ratio of the established viscosity of water over the viscosity of water in this experiment (Tang, 2016).

### Wheat Germ Agglutinin Staining

Bacterial aggregates were rinsed three times with sterile PBS, fixed with 4% paraformaldehyde, 37°C, 15 min, rinsed three times with sterile PBS, and stained with 5 μg/ml AlexaFluor ^®^488 conjugated wheat germ agglutinin (WGA; Invitrogen), 10 min, RT. Stained aggregates were rinsed three times with sterile PBS and imaged at 488 nm using a confocal laser scanning microscope (Zeiss LSM800).

### Phenol-Soluble Modulin Luminescence Assay

LAC *psm*α-lux, LAC *psm*β-lux, or LAC P3-lux strains of USA300 MRSA were inoculated (C_f_ ∼10^6^ CFU/ml) in TSB, human serum, pooled synovial fluid, or pSynF to a final volume of 5.0 ml, followed by incubation for up to 24 h (225 rpm, 37*°*C). Every 2 h, while maintaining temperature, samples were vortexed, 2 × 100 μl were removed and placed in clear bottom white plates, and luminescence was measured using a Veritas^TM^ microplate luminometer (Turner Biosystems, Sunnyvale, CA, United States). In parallel, absorbance (λ = 600 nm) was measured on the same samples to determine bacterial growth (Tecan Infinite M1000 plate reader). Luminescent values were normalized to the absorbance at 600 nm and expressed as relative luminescent units (RLUs). Each point was determined in duplicate in a minimum of three independent experiments.

### Statistics

Statistical significance was determined using the Student’s *t*-test or the Mann–Whitney *U*-test for simple comparisons. More complex comparisons were analyzed by one-way or two-way ANOVA with a Tukey’s *post hoc* test. Alpha values were set to 0.05, and the statistical analyses were performed using SPSS IBM ^®^ Statistics Version 26 or Prism GraphPad 9.0.1.

## Results

### *Staphylococcus aureus* Aggregates in Human Synovial Fluid Are Rich in Fibrinogen and Albumin

**FIGURE 1:**
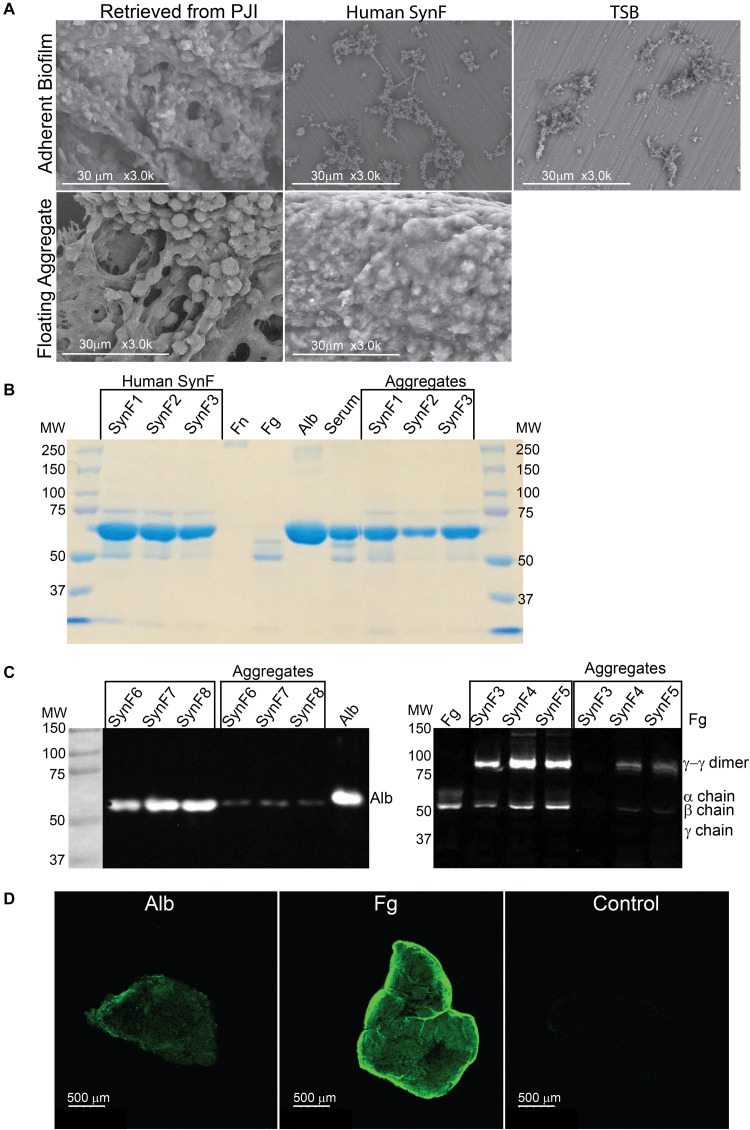
Synovial fluid aggregate morphology and composition. **(A)** **(Top)** Using SEM, biofilm morphology in samples retrieved from an infected knee replacement [periprosthetic joint infection (PJI)] or formed on titanium using methicillin-susceptible *Staphylococcus aureus* (MSSA) in human synovial fluid (SynF) or Trypticase Soy Broth (TSB). **(Bottom)** Floating aggregates retrieved from an infected knee replacement or floating aggregates formed with MSSA in human synovial fluid. **(B)** Sodium dodecyl sulfate–polyacrylamide gel electrophoresis (SDS-PAGE) gel stained with Coomassie Blue. The gel shows the composition of human synovial fluid and of synovial fluid MSSA aggregates from three separate samples of synovial fluid, as compared to fibronectin (Fn), fibrinogen (Fg), albumin (Alb), and serum controls. **(C)** Western blot analysis of MSSA aggregates from synovial fluid for Alb **(left)** and for Fg **(right)**. The γ−γ dimer and α chain are indicated, as compared with human synovial fluid, Fg, and Alb standards. Lanes represent human synovial fluid samples and resulting aggregates from different samples. **(D)** MSSA aggregates stained for Alb, Fg, or secondary antibody alone (Control). All images are representative images of at least three samples.

We started our investigation by asking if the aggregates and biofilms formed *in vitro* in human synovial fluid approximated the aggregates formed in an infected joint. By SEM (Figure 1A; all *in vitro* images are representative of at least three samples), the adherent biofilm retrieved from an implant in a case of clinically confirmed PJI was rich in proteins and even showed the presence of red blood cells. *In vitro*, MSSA biofilm formed in human synovial fluid on smooth titanium alloy (Ti6Al4V) was relatively sparse and much simpler than the PJI biofilm. The major similarity was that bacteria/biofilm showed clear fibers within and between biofilm clusters. In TSB, MSSA was largely clustered and covered by biofilm; any underlying fibrils were not as apparent as on Ti6Al4V. The non-adherent aggregate retrieved from the PJI fluid (clinical aggregate) showed large globular bacterial aggregates immobilized on a fibrous matrix and encased in biofilm. Like the PJI aggregate, large, globular, floating MSSA aggregates were formed *in vitro* in human synovial fluid from a single donor; individual bacteria were also visible and many of these were obscured by biofilm coating (Figure 1A). Because MSSA does not aggregate in TSB, no clear picture was able to be taken.

Based on our previous work (Dastgheyb et al., 2015b,c; Gilbertie et al., 2019), the abundant serum protein Fg was likely an important component of aggregates. We asked if we could identify the most abundant proteins in the aggregate, if they included Fg among other proteins, and if protein abundance was similar to the parent synovial fluid. Based on electrophoretic fractionation, the size distribution and relative amounts of human synovial fluid proteins from individual donors (SynF1, 2, 3) largely mirrored those found in human serum (Figure 1B; aggregates from the samples were fractionated on at least three gels). Proteins in the 50–75 kDa were most abundant in all samples, whereas high-MW components, which would include fibronectin (Fn), were not sufficiently abundant to be apparent with Coomassie staining of these gels. Based on staining intensity, bacterial aggregates formed in synovial fluid appeared to be composed predominantly of the same abundant proteins found in the parent synovial fluid.

Fg and Alb are both abundant in osteoarthritic synovial fluid (Fitowska et al., 2012; Mateos et al., 2012; Ritter et al., 2013). Thus, we investigated if the intense bands noted in the bacterial aggregates in Figure 1B could be associated with Fg and Alb. Both synovial fluid and their corresponding bacterial aggregates showed clear presence of Fg and Alb by Western blotting (Figure 1C). In the left-hand blot, the Alb content of three different synovial fluid samples (SynF 6, 7, 8) and their corresponding macroscopic aggregates are shown. In the right-hand blot, the Fg content of three additional human synovial fluid samples (SynF 3, 4, 5), along with the Fg content of aggregates formed in those synovial fluids, are shown. Both blots demonstrated that Alb and Fg were components of the bacterial synovial fluid aggregate. We also confirmed the presence of Fg and Alb in bacterial aggregates formed in pooled synovial fluid by immunostaining (Figure 1D). Using antibodies to the two proteins, Fg or Alb staining was clearly visible on the outside of the aggregates (in contrast to the control staining with secondary antibody; confirmed by microscopy of three aggregates). Collectively, these results established the basic components of bacterial aggregates, guiding our development of a viscous fluid for modeling bacterial behavior in synovial fluid.

### Fibrinogen Is Necessary for Bacterial Aggregation in Human Synovial Fluid

**FIGURE 2:**
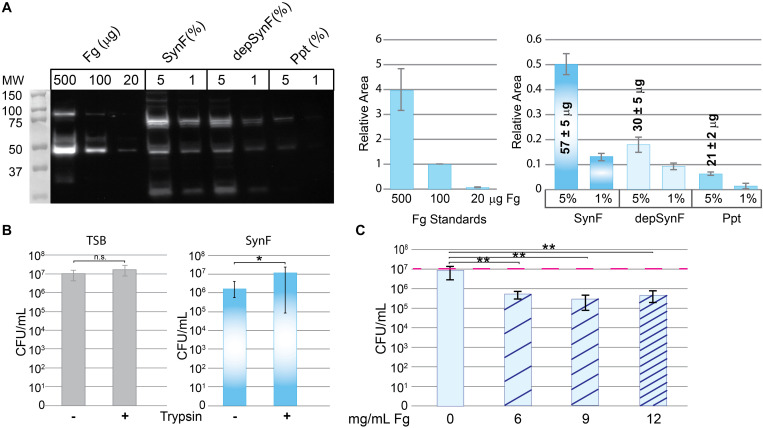
Effects of fibrinogen (Fg) on aggregation. **(A)** Western blot showing Fg standards, amount of Fg detected in pooled human synovial fluid (equal amounts added; percentage of synovial fluid in solution indicated), and amount remaining in synovial fluid after ethanol depletion of proteins including Fg (depSynF) and amount in the Fg-enriched precipitate (ppt) (>3 blots with similar results). Densitometry of this Western blot is shown on the right using varying exposures. Fg standards are plotted in the first graph and used to calculate Fg contained in the 5% samples. Areas associated with the experimental Fg samples were determined and normalized to the 100-μg sample. **(B)** Effects of aggregation in pooled synovial fluid on colony-forming units (CFUs)/ml. Minus indicates no dispersion and reflects counts obtained by dilution and counting. Plus indicates the counts after treatment of the cultures with trypsin to disperse aggregates. Values graphed are means ± SD {*p ≤ 0.05; *n* = 9 [Trypticase Soy Broth (TSB)], 12–13 (Synovial fluid)}. **(C)** Re-addition of Fg to depSynF causes reacquisition of aggregation at 90 min, as demonstrated by decreased CFU/ml. Red dashed line indicates the initial inoculum. Values graphed are means ± SD (*p ≤ 0.05; **p ≤ 0.01; *n* = 6 for each condition; representative experiment of three with similar results for each).

We next sought to explore the involvement of Fg in bacterial aggregation. To test its role, we ethanol-precipitated proteins from pooled human synovial fluid to deplete Fg (among other proteins; *n* = 3) (Qiu et al., 2003). By Western blotting (Figure 2A), the Fg content of ethanol-precipitated human synovial fluid (depSynF) was reduced compared with the parent pooled synovial fluid; the precipitate (Ppt) had faint bands corresponding to Fg. Approximate Fg concentrations were determined by densitometric comparisons of band intensities for Fg standards with experimental bands. Precipitation depleted the Fg content of synovial fluid by ∼50%, depending on the experiment.

To be able to determine the effect of altered Fg content on bacterial aggregation, we set up a trypsin dispersal assay. Specifically, we tested the ability of trypsin to digest surface proteins and other proteinaceous components that facilitate bacterial clustering in TSB and aggregation in pooled synovial fluid [Figure 2B; *n* = 9 (TSB), 12–13 (SynF)]. MSSA that typically exists in small clusters within TSB showed no significant increase in CFU after trypsin treatment. In synovial fluid, CFU were ∼1 log lower than the measured inoculum; trypsin treatment resulted in an ∼1.5 log increase in CFU. Thus, (1) synovial fluid aggregation reduced the number of retrieved CFU by counting one large cluster as one colony, and (2) trypsin treatment dispersed the aggregates to release the bacteria from the aggregates. This trypsin dispersal resulted in numbers of CFU consistent with those measured for the same inoculum in TSB.

We asked if the Fg-depleted synovial fluid (depSynF) supported MSSA aggregation (Figure 2C). No visible aggregation was observed with depSynF, and the CFUs were approximately equal to the initial inoculum (red dashed line). Addition of 6, 9, or 12 mg/ml of Fg caused a decrease in CFU, consistent with increased aggregation. Notably, 9 and 12 mg/ml Fg caused the largest decreases. In summary, we have demonstrated that Fg is crucial for visible (macroscopic) *S. aureus* aggregation in synovial fluid.

### Combinations of Albumin, Fibrinogen, and Hyaluronic Acid Can Recreate the Aggregatory Phenotype

**FIGURE 3:**
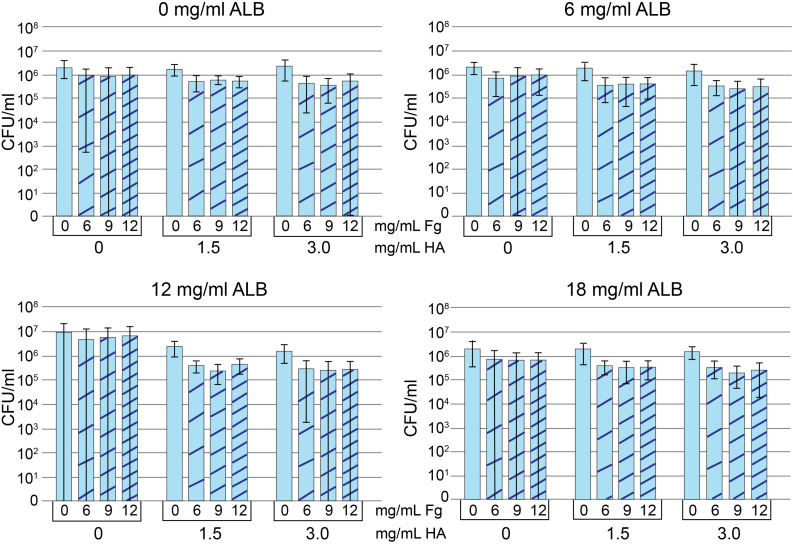
Protein addition assay. Methicillin-susceptible *Staphylococcus aureus* (MSSA) CFU/ml after growth in Trypticase Soy Broth (TSB) containing 0, 6, 12, or 18 mg/ml albumin (Alb). Increasing concentrations of hyaluronic acid (HA) and fibrinogen (Fg) are indicated in each panel. Decreased CFU/ml is a marker of increased aggregation. Values graphed are means ± SD. Statistical analyses are included in the Supplementary Material A1 (*n* = 8–9/condition).

In keeping with the presence of Fg and Alb in MSSA/MRSA aggregates, we investigated if both Fg and Alb contributed to the aggregatory antimicrobial-tolerant bacterial phenotype. Because HA contributes to the viscosity of synovial fluid (Park et al., 2014), HA was also tested. A matrix of Alb, Fg, and HA concentrations was created (Figure 3).

To measure aggregation, we looked at the dependence of component concentration on total CFU, where decreases in CFU were interpreted as increased aggregation. The addition of HA visibly increased viscosity and aggregation for all Alb, Fg concentrations/combinations. However, only 6 and 12 mg/ml Alb contained HA concentrations that were statistically different from 0 mg/ml HA (see statistical analysis in Supplementary Material A1). Among the conditions containing 6 mg/ml Alb, only 3.0 mg/ml HA (*n* = 36) resulted in lower CFU when compared to 0 mg/ml HA (*n* = 35; *p* < 0.05). When the Alb concentration was increased to 12 mg/ml, both 1.5 mg/ml (*n* = 34; *p* < 0.001) and 3.0 mg/ml HA (*n* = 36; *p* < 0.0001) resulted in greater aggregation, as manifested by lower CFU. Significantly greater aggregation occurred with (a) 12 mg/ml Alb in the presence of 1.5 or 3.0 mg/ml HA or (b) 3.0 mg/ml HA in the presence of 6 or 12 mg/ml Alb. We focused on the combination that formed the intersection of the two groups, 12 mg/ml Alb and 3.0 mg/ml HA. Addition of Fg caused some MSSA aggregation (*n* = 9; *p* < 0.001), as measured by decreased CFU. While addition of Fg always increased bacterial aggregation, a clear demarcation between the different Fg concentrations was not consistent. Ultimately, based on similarities to synovial fluid, we chose 12 mg/ml of Alb, 3 mg/ml HA, 10 mg/ml of Fg dissolved in TSB for the final concentrations. This choice was based on (a) 12 mg/ml Fg was not suitable, as it caused acquisition of a gel-like consistency to the bacteria-containing fluid (data not shown); (b) there was no difference in bacterial aggregation between 6 and 9 mg/ml Fg; and (c) known range of Fg concentrations in synovial fluid (Kushner and Somerville, 1971; Mateos et al., 2012; Bennike et al., 2014). Statistical analyses are in table form (Supplementary Material A1). We referred to this fluid as pSynF and asked if it could model bacterial behavior in human native synovial fluid.

### Methicillin-Susceptible *Staphylococcus aureus* in Pseudo Synovial Fluid Aggregate and Display Amikacin Tolerance

**FIGURE 4:**
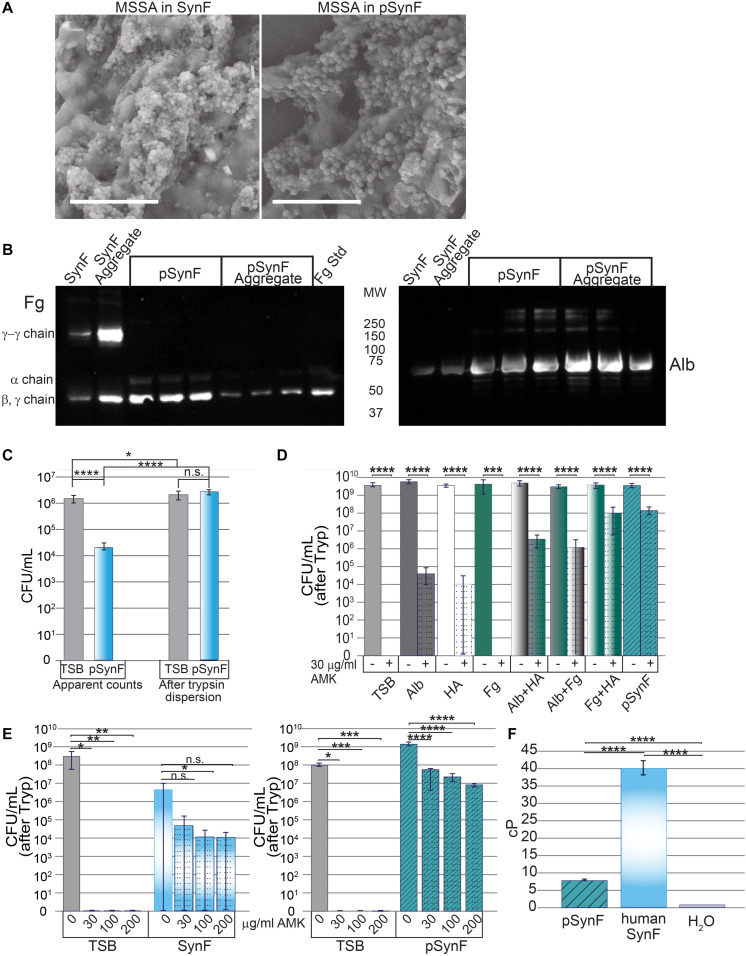
Effects of pseudo synovial fluid (pSynF) on methicillin-susceptible *Staphylococcus aureus* (MSSA). **(A)** Comparison of MSSA aggregates in human synovial fluid (SynF) vs. pSynF using SEM. Magnification: scale bar = 15 μm. **(B)** Fibrinogen (Fg) **(left)** and albumin (Alb) **(right)** content of pSynF and pSynF aggregates compared to aggregates formed in synovial fluid by Western blotting. **(C)** Graphs showing CFU/ml in Trypticase Soy Broth (TSB) and pSynF before trypsin dispersion vs. CFU/ml after trypsin dispersion [(“after Tryp” in subsequent graphs) *n* = 12 for each condition]. **(D)** Effects of amikacin (AMK) on fluids containing protein/proteoglycan concentrations found in pSynF. Controls are TSB alone and complete pSynF (*n* = 8–9 for each condition). All components were dissolved in TSB. **(E)** Dose response of MSSA to AMK in TSB vs. synovial fluid (left; *n* = 9/condition) and of TSB vs. pSynF (right; *n* = 12/condition). **(F)** Viscosity of pSynF compared to that of synovial fluid and H_2_O using a ball velocity method (*n* = 10/condition). Values graphed are means ± SD. Viscosity is expressed as centiPoise (cP). In panels **(A–F)**, *p < 0.05; **p < 0.01; ***p < 0.001; ****p < 0.0001.

To determine if the proteins contained within pSynF were able to reproduce the known characteristics of synovial fluid, we first examined the morphology of a synovial fluid aggregate compared to that of pSynF (Figure 4A). By SEM, large floating aggregates were formed in pooled synovial fluid and in pSynF. These aggregates were partially covered with a biofilm layer that obscured the finer details of the underlying aggregate; distinct fibers were only occasionally visible for either aggregate. By Western blotting, pSynF aggregates showed the presence of Fg and Alb (Figure 4B; independent replicates are shown on the blot), as was observed in the synovial fluid aggregates. The α, β, and γ Fg monomers were present in pSynF. γ-γ dimers, which were abundant in synovial fluid and synovial fluid aggregates, were sparse in the pSynF aggregates.

We next wanted to test the effects of each of the components apart and together on the antibiotic tolerance of bacteria. First, we established that, like synovial fluid, aggregation resulted in a decrease in CFU (Figure 4C; *n* = 12). Similarly, trypsin treatment caused release of MSSA from the aggregates to reach levels indistinguishable from those measured for the same inoculum in TSB.

We then tested the effect of each of the different components of pSynF when added to TSB on MSSA tolerance to AMK, an aminoglycoside antibiotic important for severe infections (Figure 4D; *n* = 8–9). In this experiment, all aggregates were dispersed by trypsin before enumeration [CFU (after Tryp)]. In TSB, 30 μg/ml of AMK eradicated MSSA (∼9 log decrease). In 12 mg/ml Alb + TSB, or 3 mg/ml HA +TSB, AMK tolerance increased so that there was only an ∼5–5.5 log decrease. It is notable that MSSA in Fg + TSB exhibited full sensitivity to AMK. MSSA in either Alb + HA + TSB or Fg + Alb + TSB showed intermediate tolerance (∼3 log decrease). MSSA exhibited the greatest tolerance to AMK in Fg + HA + TSB and the complete pSynF (HA + Fg + Alb + TSB) where 30 μg/ml AMK could only decrease MSSA levels by ∼1.5 log.

We then compared MSSA antibiotic responsiveness in pooled synovial fluid to that in pSynF (Figure 4E). In TSB, MSSA was eradicated at all doses of AMK tested. In synovial fluid, 2–3 log decreases were measured with increasing doses of AMK, although no differences were significant presumably due to incomplete dispersion of the fibrous aggregates resulting in a large variance. Increasing AMK levels in pSynF caused dose-dependent 1.5–2.5 log decreases in MSSA CFU/ml (*p* < 0.001).

To complete these analyses, we measured the viscosity of pSynF, an important property of the proteoglycan-rich synovial fluid. While the viscosity of the pSynF (∼7.5 cP) was much greater than that of water (∼1 cP), it was lower than the viscosity of human synovial fluid, which was measured as ∼40 cP in our system (Figure 4F). Collectively, these results indicate that pSynF manifests similar bacterial aggregation and antibiotic tolerance abilities to those seen in human synovial fluid.

### Virulence Factor Expression in Pseudo Synovial Fluid and Synovial Fluid

**FIGURE 5:**
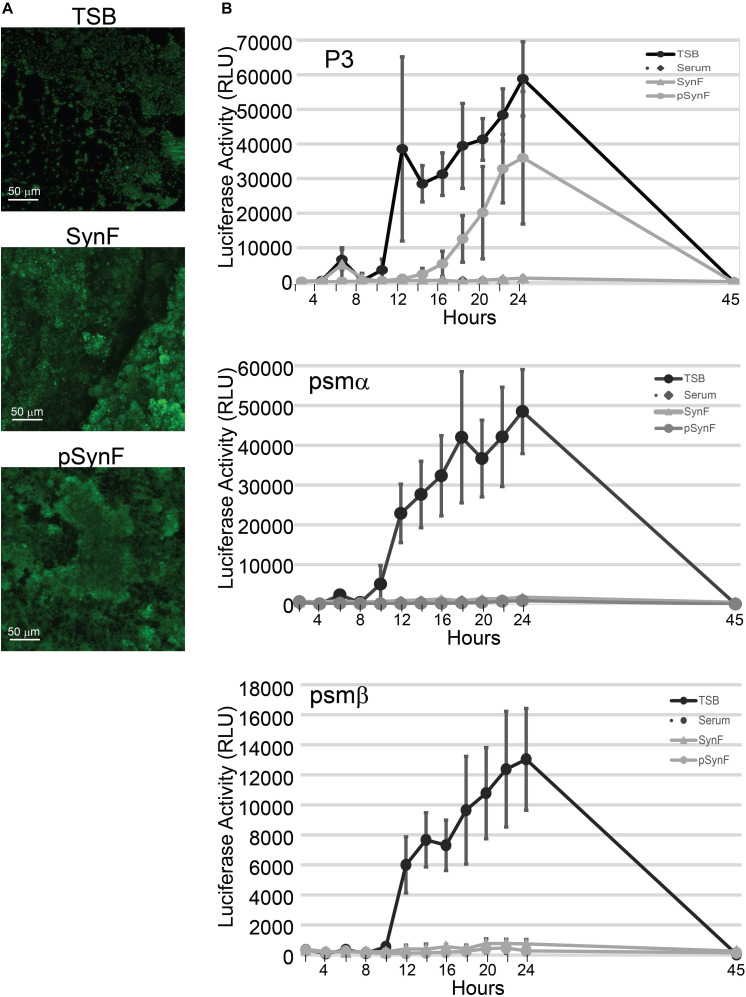
Biofilm and virulence factor expression as a function of medium. **(A)** Comparison of wheat germ agglutinen staining of polysaccharide intercellular adhesin (PIA) in methicillin-susceptible *Staphylococcus aureus* (MSSA) grown in Trypticase Soy Broth (TSB), pooled synovial fluid, or pseudo synovial fluid (pSynF). **(B)** Expression of luciferase under control of P3, *psmα*, or *psmβ* promoters using MRSA LAC300 strains in TSB, serum, pooled synovial fluid, and pSynF. Each line represents the average of three independent experiments performed in duplicate. Values graphed are means ± SD. Statistics are available in Supplementary Material A2.

Previously, we showed that both MSSA and MRSA aggregates, i.e., floating biofilms, formed in synovial fluid were covered with a polysaccharide intercellular adhesin (PIA) matrix (Dastgheyb et al., 2015a,b,c; Gilbertie et al., 2019). We thus examined WGA staining of the biofilm matrix (Figure 5A). Staining was sparse in bacteria grown in TSB. WGA staining was more intense in both synovial fluid (pooled) and pSynF and similar in intensity, suggesting similar levels of PIA production.

*S. aureus* virulence factor expression, which is under the control of the *agr* operon, is also highly influenced by the microenvironment. We have previously shown that virulence factor production in *S. aureus* is downregulated in synovial fluid (Dastgheyb et al., 2015c). We therefore sought to measure expression levels of virulence factors under the control of agr, PSMs alpha and beta, as well as the P3 promoter. The RLUs of MRSA LAC USA300 strains that expressed luciferase under control of *psmα*, *psmβ*, or P3 were determined by normalization of the measured luminescence with the corresponding OD of the growing cultures in TSB, serum, pooled synovial fluid, and pSynF (Figure 5B; *n* = 6). P3 luc expression in TSB showed a rapid and marked rise over time, whereas the expression in serum and synovial fluid was only minimally detectable. However, P3 luc expression in pSynF fell between that of synovial fluid and TSB, despite the presence of aggregation. For both *psmα* and *psmβ*, luc expression in TSB showed a marked steep rise over time, similar to the expression patterns of P3 in TSB. As we had observed previously, both serum and synovial fluid showed very low levels of *psmα* and *psmβ* luc activity. Importantly, *psmα* and *psmβ* luc expression patterns in pSynF were very similar to those measured for serum and synovial fluid.

## Discussion

We have suggested that the phenotype of bacteria in the joint directly impacts the ability of antimicrobials to combat PJI, a devastating complication of joint replacements. In previous work, we showed that synovial fluid, the lubricious fluid within the joint space, causes aggregation of many bacterial species, including MSSA and MRSA. Furthermore, we established that proteinase K and tissue plasminogen activator (tPA), presumably through activation of the serine protease plasmin, can inhibit aggregate formation, disperse the floating MSSA or MRSA aggregates formed in synovial fluid, and restore antibiotic sensitivity (Dastgheyb et al., 2015a,b; Gilbertie et al., 2019). These observations were given further rigor by examining MRSA USA300 strains from the Nebraska transposon bank, with which we previously showed (Dastgheyb et al., 2015b) that macroscopic aggregation could be abrogated in strains that had insertions in fibronectin-binding proteins A and B (FnbA and FnbB), the fibrinogen-binding Clumping factor A and B (ClfA and ClfB), and RsbU and TRAP, which are regulatory proteins (Dastgheyb et al., 2015c). Others have suggested a role for Fg and Fn in this aggregation (Walker et al., 2013; Pestrak et al., 2020). In this study, we characterized the protein content of these aggregates and set about testing which components of these aggregates were critical for this bacterial association. MSSA aggregation was dependent on increased viscosity, Alb, and Fg. We used both MSSA and MRSA strains in this work as their aggregation behavior is similar and importantly, so that our measurements of virulence factor production could be compared with our previously published work in synovial fluid (Dastgheyb et al., 2015c). Ultimately, we formulated a pSynF that could be used to study this aggregation and its effects on bacterial virulence factor production.

In the infected joint, macroscopic mucinous aggregates are routinely present and have morphological similarities to the aggregates formed *in vitro* in synovial fluid. While these aggregates show many normal components of a biofilm, based on our earlier work, the degradation of PIA or extracellular DNA is insufficient to disrupt the aggregates – a proteinase is required (Dastgheyb et al., 2015b). We thus set out to examine the components of synovial fluid aggregates. Synovial fluid is a filtrate of human serum and is enriched with proteoglycans produced by synoviocytes and chondrocytes. We used both individual and pooled samples of human synovial fluid, with similar results on aggregation, protein composition, and antibiotic tolerance. Unlike synovial fluid, serum aggregates tend to be small except in end-stage sepsis (Iskander et al., 2013). *In vitro*, aggregation in serum, albeit microscopic, does occur (Walker et al., 2013; Dastgheyb et al., 2015a), but it is worth noting that laboratory serum has had Fg precipitated or cross-linking inhibited during the process of collection and isolation. Taken together, we thought that Fg and perhaps proteoglycans, specifically HA, would be necessary for recapitulating the aggregatory phenotype. In fact, others have shown that Fg and Fn can cause bacterial aggregation when added to ideal media (Pestrak et al., 2020), which is consistent with our earlier experiments with aggregate dispersion.

Using aggregates from three independent human synovial fluid samples, the aggregate protein content and concentrations were determined to be similar to those of the parent synovial fluid by SDS-PAGE. Furthermore, the apparent protein distribution mirrored the pattern of human serum (within the limits of Coomassie blue staining) (Lyngbye and Krøll, 1971). Based on known proteins in serum and synovial fluid and their relative abundance (Ritter et al., 2013; Bennike et al., 2014), we identified Alb and Fg as major components of the aggregate, with a number of minor components that we did not identify. While our previous work and that of others using tPA suggested a critical role for Fg (Dastgheyb et al., 2015a,b; Gilbertie et al., 2019; Pestrak et al., 2020), only through Fg complementation could its role be established. Thus, we depleted pooled synovial fluid of Fg by ethanol precipitation of proteins. Nevertheless, the ethanol precipitation, while enriching the precipitate for Fg, precipitated other proteins that could also play a role in the bacterial response to synovial fluid, e.g., aggregation. However, addition of Fg back to the ethanol-precipitated synovial fluid restored visible aggregation and supported its proposed role, in keeping with our published work and work by others (Walker et al., 2013; Dastgheyb et al., 2015a,b,c; Pestrak et al., 2020). Bacterial binding to these proteins would use the MSCRAMMs that, through use of MRSA USA300 strains from the Nebraska transposon bank, we previously showed moderated macroscopic aggregation (Dastgheyb et al., 2015b). Specifically, aggregation could be abrogated in strains that had insertion mutations in FnbA and FnbB, the ClfA and ClfB, prominent MSCRAMMs, and RsbU and TRAP, which are regulatory proteins (Dastgheyb et al., 2015c).

Interestingly, Alb and HA were also important for aggregation, as demonstrated in the combinatorial addition experiments. Within the range of 1.5–3.0 mg/ml HA, 6–12 mg/ml Alb, and 6–12 mg/ml Fg, MSSA and MRSA aggregated, as evidenced by decreased CFU. Furthermore, if only one of the components was present, the ability to reproduce the characteristic antibiotic tolerance was attenuated. Interestingly, antibiotic tolerance measured in HA + Fg + TSB was similar to that in pSynF, despite the dependence on Alb that aggregation was shown to have. It is interesting that the Fg concentration showed an upper limit as the 12 mg/ml Fg concentrations started to become more gel-like (rather than distinct aggregates) in the presence of MSSA. Importantly, we chose the concentrations of the different components based on statistical tests using aggregation, antibiotic tolerance, and ultimately literature values for human synovial fluid (which fell within the ranges we had determined) (Mateos et al., 2012; Bennike et al., 2014).

The proteins sequestered within the MSSA aggregates formed in pSynF approximated the protein composition of that in human synovial fluid, although individual and cross-linked chains of Fg were not always equivalent in the pSynF vs. synovial fluid aggregates, perhaps pointing to the absence of the Fg cross-linking cascade in pSynF. When aggregates were viewed without magnification, the pSynF aggregates were uniformly smaller than those formed in synovial fluid and in clinical cases where we have observed retrieval of more mucinous aggregates. It is of course probable that other proteins will have roles in subtly modifying the stability, antibiotic sensitivity, and morphology of the aggregates. We would suggest either the localization of additional proteoglycans found in synovial fluid or greater production of PIA, as well as perhaps additional proteins, that modifies the density of the aggregate. Possibilities would include collagen that binds to the same MSCRAMMs previously identified and Fn, a protein that decorates most extracellular matrices; all are present in synovial fluid. Interestingly, the Fg and Alb content of synovial fluid increases in the osteoarthritic joint (Mateos et al., 2012; Bennike et al., 2014). We similarly expect an increase in the infected joint, making them more available for bacterial aggregation. Nevertheless, both human synovial fluid and pSynF conferred marked antibiotic tolerance to MSSA aggregates, with similar decreases when compared to the untreated control.

While clinically relevant joint-lubricating fluids (Adams et al., 1995; Neustadt et al., 2005; Mathies, 2006; Petterson and Plancher, 2019), as well as those for the study of rheology (Radin et al., 1971; Takadama and Mizuno, 2006; Bortel et al., 2015), have been formulated, our goal was to study bacterial responses to synovial fluid proteins. Interestingly, the viscosity of pSynF, while ∼7× that of water, was still lower than that which we measured for native synovial fluid, even though the concentrations of HA tested were informed by known amounts of HA in synovial fluid. A number of proteoglycans produced in the joint capsule contribute to this viscosity, including the MW of the HA itself and an important role played by the lubricin macromolecule (Jay et al., 2007). Importantly, despite the difference in viscosity, both pSynF and synovial fluid caused marked antibiotic tolerance. An important role for viscosity/polarity of the medium was indicated by the requirement for HA to obtain the full antibiotic tolerance associated with Fg or Fg + Alb. The importance, however, of understanding the underpinnings of this antibiotic tolerance remains focused on restoring antibiotic susceptibility, especially as the mechanism is likely to be the same poorly defined bacterial metabolism and protein expression changes that characterize the biofilm phenotype. By defining the importance of the three components within pSynF, we also define targets for therapeutic interventions. One such possibility already exists with our reports of tPA dispersal (and restoration of antibiotic susceptibility) to the MSSA aggregates.

Further emphasizing the similarity of these aggregates to biofilms, both synovial fluid and pSynF formed PIA-encased aggregates that were similar in morphology and apparent intensity of WGA staining. Furthermore, expression levels of PSM-α and -β reporter plasmids suggested that Fg, Alb, and HA are key mediators of bacterial behavior in synovial fluid, especially as similar magnitudes and kinetics of expression were noted in serum, synovial fluid, and pSynF. However, it is not clear if the lower viscosity, or perhaps the use of TSB as the reconstituting medium, led to a difference in P3 expression by MRSA USA300. Finally, we used P3 expression as a readout of Agr regulation. As Agr regulates quorum sensing, as well as the expression of a subset of virulence factors (Dastgheyb and Otto, 2015), it becomes important to elucidate the additional components that suppress P3 expression and, by implication, Agr activity in human physiological fluid. Finally, we would suggest that the overall reproduction of aggregation, biofilm slime, antibiotic resistance, and PSM expression suggests that these three components are critical mediators of bacterial phenotype and in the process furnish an important tool to determine bacterial/biomaterial interactions in a system that more faithfully reproduces the *in vivo* environment.

In summary, we have identified Fg, Alb, and HA as critical components responsible for bacterial aggregation in synovial fluid and that together, as presented in pSynF, these proteins interact with MSSA and MRSA to recapitulate the majority of the properties of MSSA or MRSA in synovial fluid aggregates. Importantly, as we have previously shown, both serum and synovial fluid cause marked decreases in virulence factor expression, in contrast to many studies suggesting an apparent increase in Agr-controlled genes associated with infections (Le and Otto, 2015). Our studies emphasize the importance of using physiological and model physiological fluids such as this pSynF in studying bacteria–biomaterial interactions. This formulated pSynF can, in fact, be used as a reasonable alternative for studying *in vitro* models of PJI and septic arthritis, as well as providing a more realistic medium for the development of antimicrobial strategies/materials for implants.

## Data Availability Statement

The raw data supporting the conclusions of this article will be made available by the authors, without undue reservation.

## Author Contributions

SK and DC contributed to the methodology, formal analysis, investigation, writing, review, and editing, visualization, and writing the final approved version. NZ contributed to the validation, formal analysis, investigation, writing, review, and editing, and writing the final approved version. PM contributed to the methodology, investigation, visualization, and writing the final approved version. SD contributed to the methodology, investigation, writing, review, and editing, and writing the final approved version. CP contributed to the validation, investigation, writing, review, and editing, and writing the final approved version. MH and AC contributed to the resources, writing, review, and editing, and writing the final approved version. TS contributed to the conceptualization, resources, writing, review, and editing, funding acquisition, and writing the final approved version. MO contributed to the methodology, resources, writing, review, and editing, visualization, funding acquisition, and writing the final approved version. NH contributed to the conceptualization, writing the original draft, writing, review, and editing, visualization, supervision, funding acquisition, and writing the final approved version. All authors contributed to the article and approved the submitted version.

## Conflict of Interest

The authors declare that the research was conducted in the absence of any commercial or financial relationships that could be construed as a potential conflict of interest.
