# Supplementary material for: Staphylococcus aureus Floating Biofilm Formation and Phenotype in Synovial Fluid Depends on Albumin, Fibrinogen, and Hyaluronic Acid
Source: Front Microbiol. 2021 Apr 29;12:655873. doi: 10.3389/fmicb.2021.655873 (PMC8117011; doi:10.3389/fmicb.2021.655873)
Supplement: Supplementary file 1 [file Data_Sheet_1.PDF]

Supplemental Material A1: Statistical reasoning testing effects of HA, Fg, and Alb, Figure 3

A. Comparing 0, 1.5, and 3.0 mg/ml HA concentration groups with a fixed concentration of ALB to determine which concentrations are different from 0 for each given ALB concentration. Each table of comparisons is followed by a table comparing between group effects using a 2-way ANOVA with the Tukey post hoc test for multiple comparisons.

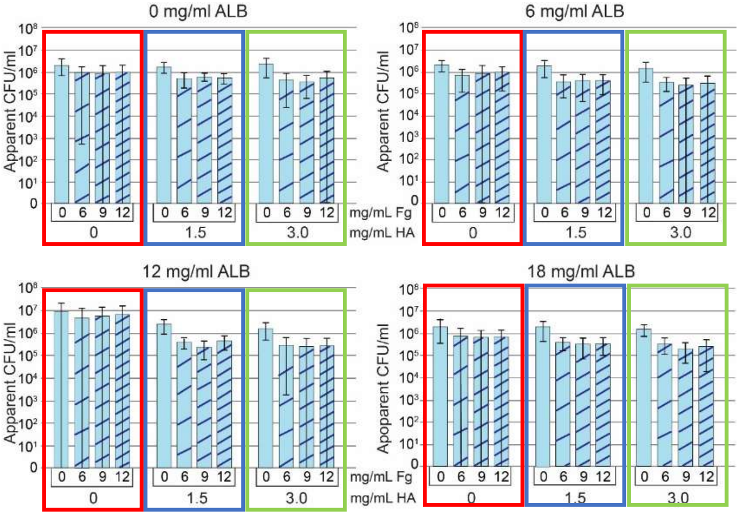

Statistical tables:

0 mg/ml ALB, comparing HA groups
Multiple Comparison

Dependent Variable: VAR0001						
Tukey HSD						
(I) HA	(J) HA	Mean Difference (I-J)	Std. Error	Sig.	95% Confidence Interval	
					Lower Bound	Upper Bound
0 mg/ml	1.5 mg/ml	345060.0000	205025.2000	0.217	-143271.7164	833391.7164
	3.0 mg/ml	271780.0000	205025.2000	0.385	-216551.7164	760111.7164
1.5 mg/ml	0 mg/ml	-345060.0000	205025.2000	0.217	-833391.7164	143271.7164
	3.0 mg/ml	-73280.0000	205025.2000	0.932	-561611.7164	415051.7164
3.0 mg/ml	0 mg/ml	-271780.0000	205025.2000	0.385	-760111.7164	216551.7164
	1.5 mg/ml	73280.0000	205025.2000	0.932	-415051.7164	561611.7164
Based on observed means. The error term is Mean Square(Error)=735618321014.038						

n = 35 (HA 0 mg/ml); 35 (HA 1.5 mg/ml); 35 (HA 3.0 mg/mL)

Tests of Between-Subjects Effects

Dependent Variable = CFU/ml					
Source	Type III Sum of Squares	df	Mean Square	F	Sig
Corrected Model	4.121E+13[a]	11	3.746E+12	5.093	0.000
Intercept	9.234E+13	1	9.234E+13	125.521	0.000
HA	2.306E+12	2	1.153E+12	1.567	0.214
Fg	3.669E+13	3	1.223E+13	16.627	0.000
HA*Fg	2.214E+12	6	3.690E+11	0.502	0.806
Error	6.841E+13	93	7.356E+11		
Total	2.041E+14	105			
Corrected Total	1.096E+14	104			
[a]R squared = 0.376 (Adjusted R squared = 0.302)					

6 mg/ml ALB, comparing HA groups
Multiple Comparison

Dependent Variable: VAR0001						
Tukey HSD						
(I) HA	(J) HA	Mean Difference (I-J)	Std. Error	Sig.	95% Confidence Interval	
					Lower Bound	Upper Bound
0 mg/ml	1.5 mg/ml	379188.8889	183563.4230	0.102	-57803.3970	816181.1747
	3.0 mg/ml	547518.6111*	183563.4230	0.010	110526.3253	984510.8970
1.5 mg/ml	0 mg/ml	-379188.8889	183563.4230	0.102	-816181.1747	57803.3970
	3.0 mg/ml	168329.7222	183563.4230	0.631	-268662.5636	605322.0081
3.0 mg/ml	0 mg/ml	-547518.611*	183563.4230	0.010	-984510.8970	-110526.3253
	1.5 mg/ml	-168329.7222	183563.4230	0.631	-605322.0081	268662.5636
Based on observed means. The error term is Mean Square(error) = 606519544925.000						
*The mean difference is significant at the 0.05 level						

n = 36 (HA 0 mg/ml); 36 (HA 1.5 mg/ml); 36 (HA 3.0 mg/mL)

Tests of Between-Subjects Effects

Dependent Variable = CFU/ml					
Source	Type III Sum of Squares	df	Mean Square	F	Sig
Corrected Model	4.152E+13[a]	11	3.774E+12	6.223	0.000
Intercept	7.686E+13	1	7.686E+13	126.721	0.000
HA	5.663E+12	2	2.831E+12	4.668	0.012
Fg	3.518E+13	3	1.173E+13	19.334	0.000
HA*Fg	6.755E+11	6	1.126E+11	0.186	0.980
Error	5.823E+13	96	6.065E+11		
Total	1.766E+14	108			
Corrected Total	9.974E+13	107			
[a]R squared = 0.416 (Adjusted R squared = 0.349)					

12 mg/ml ALB, comparing HA groups

Multiple Comparison						
Dependent Variable: VAR0001						
Tukey HSD						
(I) HA	(J) HA	Mean Difference (I-J)	Std. Error	Sig.	95% Confidence Interval	
					Lower Bound	Upper Bound
0 mg/ml	1.5 mg/ml	5436143.46*	1254727.036	0.000109	2448133.648	8424153.280
	3.0 mg/ml	5730383.33*	1236672.469	0.000034	2785368.706	8675397.961
1.5 mg/ml	0 mg/ml	-5436143.46*	1254727.036	0.000109	-8424153.28	-2448133.65
	3.0 mg/ml	294239.8693	1254724.036	0.970149	-2693759.95	3282249.685
3.0 mg/ml	0 mg/ml	-5730383.33*	1236672.469	0.000034	-8675397.96	-2785368.71
	1.5 mg/ml	-294239.8693	1254727.036	0.970149	-3282249.69	2693769.947
Based on observed means. The error term is Mean Square(Error) = 27528458302387.730						
*The mean difference is significant at the 0.05 level.						

n = 36 (HA 0 mg/ml); 34 (HA 1.5 mg/ml) 36 (HA 3.0 mg/mL)

Tests of Between-Subjects Effects

Dependent Variable = CFU/ml					
Source	Type III Sum of Squares	df	Mean Square	F	Sig
Corrected Model	8.804E+14[a]	11	8.003E+13	2.907	0.002
Intercept	7.182E+14	1	7.182E+14	26.091	0.000
HA	7.471E+14	2	3.735E+14	13.569	0.000
Fg	1.117E+14	3	3.723E+13	1.352	0.262
HA*Fg	2.508E+13	6	4.180E+12	0.152	0.988
Error	2.588E+15	94	2.753E+13		
Total	4.211E+15	106			
Corrected Total	3.468E+15	105			
[a]R squared = 0.254 (Adjusted R squared = 0.167)					

18 mg/ml ALB, comparing HA groups

Multiple Comparison						
Dependent Variable: VAR0001						
Tukey HSD						
(I) HA	(J) HA	Mean Difference (I-J)	Std. Error	Sig.	95% Confidence Interval	
					Lower Bound	Upper Bound
0 mg/ml	1.5 mg/ml	290572.2222	205908.2994	0.339	-199614.4225	780758.8669
	3.0 mg/ml	431616.6667	205908.2994	0.096	-58569.9780	921803.3114
1.5 mg/ml	0 mg/ml	-290572.2222	205908.2994	0.339	-780758.8669	199614.4225
	3.0 mg/ml	141044.4444	205908.2994	0.773	-349142.2003	631231.0892
3.0 mg/ml	0 mg/ml	-431616.6667	205908.2994	0.096	-921803.3114	58569.9780
	1.5 mg/ml	-141044.4444	205908.2994	0.773	-631231.0892	349142.2003
Based on observed means. The error term is Mean Square(Error) = 763168099629.630						

n = 36 (HA 0 mg/ml); 36 (HA 1.5 mg/ml) 36 (HA 3.0 mg/mL)

Tests of Between-Subjects Effects

Dependent Variable = CFU/ml					
Source	Type III Sum of Squares	df	Mean Square	F	Sig
Corrected Model	5.104E+13[a]	11	4.640E+12	6.080	0.000
Intercept	7.875E+13	1	7.875E+13	103.190	0.000
HA	3.487E+12	2	1.744E+12	2.285	0.107
Fg	4.737E+13	3	1.579E+13	20.688	0.000
HA*Fg	1.855E+11	6	3.091E+10	0.041	1.000
Error	7.326E+13	96	7.632E+11		
Total	2.031E+14	108			
Corrected Total	1.243E+14	107			
[a]R squared = 0.411 (Adjusted R squared = 0.343)					

B. **After selecting the groups where significance occurred for the different HA concentrations, we compared** the different Fg concentrations within each HA concentration. N.B., Fg in 6 mg/ml ALB-60% HA are only compared within that group. For the Fg values in 12 mg/ml Alb, the groups in 1.5 mg/ml HA and in 3.0 mg/ml HA are compared within the group. Comparisons are performed with 1-way ANOVA

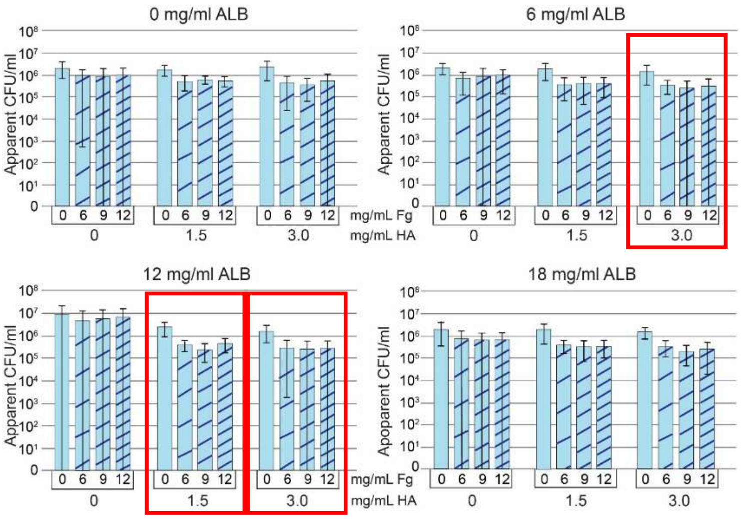

6 mg/ml ALB, 3 mg/ml HA

Multiple Comparison						
Dependent Variable: VAR0001						
Tukey HSD						
(I) Fg	(J) Fg	Mean Difference (I-J)	Std. Error	Sig.	95% Confidence Interval	
					Lower Bound	Upper Bound
0 mg/ml	6 mg/ml	1188903.33*	300138.9922	0.002	375718.2531	2002088.414
	9 mg/ml	1265392.22*	300138.9922	0.001	452207.1420	2078577.302
	12 mg/ml	1211170.00*	300138.9922	0.002	397984.9198	2024355.080
6 mg/ml	0 mg/ml	-1188903.33*	300138.9922	0.002	-2002088.41	-375718.2531
	9 mg/ml	76488.8889	300138.9922	0.994	-736696.1913	889673.9691
	12 mg/ml	22266.6667	300138.9922	1.000	-790918.4136	835451.7469
9 mg/ml	0 mg/ml	-1265392.22	300138.9922	0.001	-2078577.30	-452207.1420
	6 mg/ml	-76488.8889	300138.9922	0.994	-889673.9691	736696.1913
	12 mg/ml	-54222.2222	300138.9922	0.998	-867407.3024	758962.8580
12 mg/ml	0 mg/ml	-1211170.00	300138.9922	0.002	-2024355.08	-397984.9198
	6 mg/ml	-22266.6667	300138.9922	1.000	-835451.7469	790918.4136
	9 mg/ml	54222.2222	300138.9922	0.998	-758962.8580	867407.3024
Based on observed means. The error term is Mean square (Error) = 405375365886.111						
*The mean difference is significant at the 0.05 level						

n = 9 for each of the Fg conditions (0, 6, 9, 12 mg/ml)

12 mg/ml ALB, 1.5 mg/ml HA

Multiple Comparison						
Dependent Variable: VAR0001						
Tukey HSD						
(I) Fg	(J) Fg	Mean Difference (I-J)	Std. Error	Sig.	95% Confidence Interval	
					Lower Bound	Upper Bound
0 mg/ml	6 mg/ml	2030222.22*	402854.3528	0.000117	934817.1756	312567.269
	9 mg/ml	2172133.33*	415252.7621	0.000069	1043015.656	3301251.011
	12 mg/ml	1967383.33*	415252.7621	0.000272	838265.6558	3096501.011
6 mg/ml	0 mg/ml	-2030222.22*	402854.3528	0.000117	-3125627.27	-934817.1756
	9 mg/ml	141911.1111	415252.7621	0.985996	-987206.5664	1271028.789
	12 mg/ml	-62838.8889	415252.7621	0.998736	-1101056.57	1066278.789
9 mg/ml	0 mg/ml	-2172133.33*	415252.7621	0.000069	-3301251.01	-1043015
	6 mg/ml	-141911.1111	415252.7621	0.985996	-1271028.79	987206.5664
	12 mg/ml	-204750.0000	427291.5670	0.963115	-1366602.50	957102.5049
12 mg/ml	0 mg/ml	-1967383.33*	415252.7621	0.000272	-3096501.01	-838265.6558
	6 mg/ml	62838.8889	415252.7621	0.998736	-1066278.79	1191956.566
	9 mg/ml	204750.0000	427291.5670	0.963115	-957102.5049	1366602.505
Based on observed means. The error term is Mean Square(Error) = 730312332962.963						
*The mean difference is significant at the 0.05 level.						

n = 9 (Fg 0 mg/ml); 9 (Fg 6 mg/ml); 8 (Fg 9 mg/ml); 9 (Fg 12 mg/ml)

12 mg/ml ALB, 3 mg/ml HA

Multiple Comparison						
Dependent Variable: VAR0001						
Tukey HSD						
(I) Fg mg/ml	(J) Fg mg/ml	Mean Difference (I-J)	Std. Error	Sig.	95% Confidence Interval	
					Lower Bound	Upper Bound
0	6	1325733.33*	299408.8785	0.000575	514526.3951	2136940.272
	9	1356000.00*	299408.8785	0.000432	544793.0618	2167206.938
	12	1352088.89*	299408.8785	0.000449	540881.9507	2163295.827
6	0	-1325733.33*	299408.8785	0.000575	-2136940.27	-514526.3951
	9	30266.6667	299408.8785	0.999622	-780940.2716	841473.6049
	12	26355.5556	299408.8785	0.999750	-784851.3827	837562.4938
9	0	-1356000.00*	299408.8785	0.000432	-2167206.94	-544793.0618
	6	-30266.6667	299408.8785	0.999622	-841473.6049	780940.2716
	12	-3911.1111	299408.8785	0.999999	-815118.0493	807295.8271
12 mg/ml	0	-1352088.89*	299408.8785	0.000449	-2163295.83	-540881.9507
	6	-26355.5556	299408.8785	0.999750	-837562.4938	784851.3827
	9	3911.1111	299408.8785	0.999999	-807295.8271	815118.0493
Based on observed means. The error term is Mean Square(Error) = 403405544444.445						
*The mean difference is significant at the 0.05 level.						

n = 9 (Fg 0 mg/ml); 9 (Fg 6 mg/ml); 9 (Fg 9 mg/ml); 9 (Fg 12 mg/ml)

Supplemental Material A2: Statistical reasoning testing effects of HA, Fg, and Alb, Figure 5

Table S2: The purpose of the experiments was to test whether pSynF caused similar effects on gene expression as SynF. The indicated comparison is boxed in blue. Within that blue row, statistical significance of $p<0.05$ is shown without highlighting, whereas $p>0.05$, i.e., no apparent significant difference is highlighted in grey. pSynF is compared to SynF to see if the protein content in pSynF causes the same gene regulation, as a function of promoter, as that in intact SynF. While most values indicate significant differences in P3 expression over time, expression levels associated with *psmα* and *psmβ* appear similar between pSynF and SynF, suggesting equivalent gene regulation.

**P3**													
	**Time (Hours)**	**2**	**4**	**6**	**8**	**10**	**12**	**14**	**16**	**18**	**20**	**22**	**24**
**p-value**	**TSB vs Serum**	<0.001	0.001	0.999	0.935	ns	0.117	0.956	0.984	0.992	0.998	0.982	0.994
	**TSB vs SynF**	<0.001	<0.001	<0.001	0.013	ns	0.094	0.878	0.964	1	0.998	0.981	0.991
	**TSB vs pSynF**	0.053	0.02	1	0.937	ns	<0.001	<0.001	<0.001	<0.001	<0.001	<0.001	<0.001
	**Serum vs SynF**	0.263	<0.001	<0.001	0.004	ns	0.95	0.988	0.998	0.998	1	1	1
	**Serum vs pSynF**	0.08	0.985	0.999	0.716	ns	0.002	<0.001	<0.001	<0.001	<0.001	<0.001	<0.001
	**SynF vs pSynF**	0.004	<0.001	<0.001	0.109	ns	0.02	<0.001	<0.001	<0.001	<0.001	<0.001	<0.001
**ANOVA p-value**		<0.001	<0.001	<0.001	0.005	0.697	<0.001	<0.001	<0.001	<0.001	<0.001	<0.001	<0.001

psmα													
	**Time (Hours)**	**2**	**4**	**6**	**8**	**10**	**12**	**14**	**16**	**18**	**20**	**22**	**24**
**p-value**	**TSB vs Serum**	<0.001	0.86	<0.001	<0.001	<0.001	<0.001	<0.001	<0.001	<0.001	<0.001	<0.001	<0.001
	**TSB vs SynF**	0.001	<0.001	<0.001	0.015	0.007	<0.001	<0.001	<0.001	<0.001	<0.001	<0.001	<0.001
	**TSB vs pSynF**	0.279	0.947	<0.001	<0.001	0.006	<0.001	<0.001	<0.001	<0.001	<0.001	<0.001	<0.001
	**Serum vs SynF**	<0.001	<0.001	0.532	0.002	0.999	0.998	0.998	0.997	1	0.999	1	0.998
	**Serum vs pSynF**	<0.001	0.683	0.997	0.996	1	0.999	0.999	1	1	1	1	1
	**SynF vs pSynF**	0.194	<0.001	0.753	0.002	0.997	0.994	0.995	0.993	1	0.997	1	0.998
**ANOVA p-value**		<0.001	<0.001	<0.001	<0.001	<0.001	<0.001	<0.001	<0.001	<0.001	<0.001	<0.001	<0.001

psmβ													
**Time (Hours)**		**2**	**4**	**6**	**8**	**10**	**12**	**14**	**16**	**18**	**20**	**22**	**24**
**p-value**	**TSB vs Serum**	<0.001	ns	<0.001	0.107	<0.001	<0.001	<0.001	<0.001	<0.001	<0.001	<0.001	<0.001
	**TSB vs SynF**	0.311	ns	0.003	<0.001	<0.001	<0.001	<0.001	<0.001	<0.001	<0.001	<0.001	<0.001
	**TSB vs pSynF**	0.004	ns	0.325	0.005	<0.001	<0.001	<0.001	<0.001	<0.001	<0.001	<0.001	<0.001
	**Serum vs SynF**	<0.001	ns	0.992	<0.001	1	0.998	1	0.973	1	0.995	0.994	0.988
	**Serum vs pSynF**	0.891	ns	0.099	0.31	0.869	0.997	0.996	0.996	1	1	1	0.994
	**SynF vs pSynF**	<0.001	ns	0.28	0.028	0.922	0.998	0.994	0.944	1	0.996	0.999	0.959
**ANOVA p-value**		<0.001	0.786	<0.001	<0.001	<0.001	<0.001	<0.001	<0.001	<0.001	<0.001	<0.001	<0.001
